# Characterization of the human *DYRK1A *promoter and its regulation by the transcription factor E2F1

**DOI:** 10.1186/1471-2199-9-30

**Published:** 2008-03-26

**Authors:** Barbara Maenz, Paul Hekerman, Eva M Vela, Juan Galceran, Walter Becker

**Affiliations:** 1 Institute of Pharmacology and Toxicology, Medical Faculty of the RWTH Aachen University, Wendlingweg 2, 52074 Aachen, Germany; 2 Instituto de Neurociencias, CSIC – Universidad Miguel Hernandez, Campus de San Juan, 03550 San Juan (Alicante), Spain

## Abstract

**Background:**

Overexpression of the human *DYRK1A *gene due to the presence of a third gene copy in trisomy 21 is thought to play a role in the pathogenesis of Down syndrome. The observation of gene dosage effects in transgenic mouse models implies that subtle changes in expression levels can affect the correct function of the *DYRK1A *gene product. We have therefore characterized the promoter of the human *DYRK1A *gene in order to study its transcriptional regulation.

**Results:**

Transcription start sites of the human *DYRK1A *gene are distributed over 800 bp within a region previously identified as an unmethylated CpG island. We have identified a new alternative noncoding 5'-exon of the *DYRK1A *gene which is located 772 bp upstream of the previously described transcription start site. Transcription of the two splicing variants is controlled by non-overlapping promoter regions that can independently drive reporter gene expression. We found no evidence of cell- or tissue-specific promoter usage, but the two promoter regions differed in their activity and their regulation. The sequence upstream of exon 1A (promoter region A) induced about 10-fold higher reporter gene activity than the sequence upstream of exon 1B (promoter region B). Overexpression of the transcription factor E2F1 increased *DYRK1A *mRNA levels in Saos2 and Phoenix cells and enhanced the activity of promoter region B three- to fourfold.

**Conclusion:**

The identification of two alternatively spliced transcripts whose transcription is initiated from differentially regulated promoters regions indicates that the expression of the *DYRK1A *gene is subject to complex control mechanisms. The regulatory effect of E2F1 suggests that DYRK1A may play a role in cell cycle regulation or apoptosis.

## Background

Protein kinases of the DYRK (dual specificity tyrosine phosphorylation-regulated kinase) family play key roles in the regulation of cell growth and differentiation in a variety of systems [1,2]. Processes controlled by DYRKs include the organization of the symmetric cell division in *S. pombe *[3], the transition from growth to development in *Dictyostelium *[4], the formation of the embryonic axis in *C. elegans *[5], and the control of erythropoiesis in mammals [6]. In *Drosophila*, the kinase encoded by the *minibrain *gene (MNB) plays an essential role in postembryonic neurogenesis [7], and the orthologous proteins in chicken (MNB) and mammals (DYRK1A) have also been implicated in the regulation of neuronal differentiation (reviewed in [8]). Because the human *DYRK1A *gene is localized within a region of chromosome 21 considered to be particularly important for many traits of Down syndrome ("Down syndrome critical region") [9,10], *DYRK1A *has attracted interest as a candidate gene for brain abnormalities and mental retardation in individuals with Down syndrome.

The proposed role of DYRK1A in Down syndrome-related mental retardation was supported by analyses of genetically altered mice. Three different mouse models (transgenic mice with a yeast artificial chromosome that includes the human *DYRK1A *gene, transgenic mice overexpressing the cDNA of rat *Dyrk1a*, and transgenic mice with one extra copy of the human *DYRK1A *gene in a bacterial artificial chromosome) were generated and found to exhibit neurodevelopmental delays and impairment in learning tasks [11-13]. Interestingly, mice heterozygous for *Dyrk1a *also show marked abnormalities of brain development and behavior [14-17], providing evidence that the function of DYRK1A is particularly sensitive to gene dosage effects. It is generally thought that the many features of Down syndrome originate from a 1.5-fold increase in the dosage of genes in the Down syndrome critical region [18]. Expression of the human *DYRK1A *was shown to be increased 1.5-fold in fetal and adult brains from subjects with Down syndrome [19-21].

These results imply that the function of DYRK1A is strongly influenced by its level of expression. The activity of many protein kinases is subject to short term control by second messengers (Ca^2+^, cAMP, AMP), posttranslational modifications such as phosphorylation, or interaction with regulatory subunits (cyclins, CDK). In contrast, DYRKs require phosphorylation of a conserved tyrosine residue in the activation loop to acquire maximal catalytic activity, but this is an autophosphorylation event that takes place during maturation of the protein and does not appear to be subject to regulation [22,23]. Although the activity of DYRK1A has been reported to be modulated by bFGF (basic fibroblast growth factor) [24] and by interaction with 14-3-3 proteins [25], the kinase was found to be constitutively active in all systems studied so far. Hence, regulation of gene expression will directly influence the cellular function of DYRK1A. Microarray studies have revealed striking changes in the abundance of *DYRK1A *mRNA in various systems of cellular differentiation and proliferation, *e.g*., during activation of T-cells [26], in human neutrophils exposed to bacteria [27], in differentiating haematopoetic progenitor cells [28], in the course of melanoma tumor progression [29], or in HPV16-immortalized keratinocytes [30]. Interestingly, expression of DYRK1A is also tightly controlled in neural progenitor cells of early developing chicken brains [31]. Altogether, these studies clearly indicate that *DYRK1A *mRNA levels are highly regulated, although the question how the expression of the *DYRK1A *gene is controlled has not been directly addressed until now.

In this study we have characterized the promoter of the human *DYRK1A *gene in order to analyze its transcriptional regulation. Our data show that transcription of the *DYRK1A *gene can start within a broad region of 800 bp. Two non-overlapping regions within the 5'-region of the *DYRK1A *gene exhibit promoter activity in reporter gene assays and lead to transcripts with alternative noncoding 5' exons. Interestingly, one of the two promoter regions is regulated by the transcription factor E2F1, suggesting a potential role of DYRK1A in the cell cycle.

## Results

### Alternative transcription start sites in the human *DYRK1A *gene

**Figure 1:**
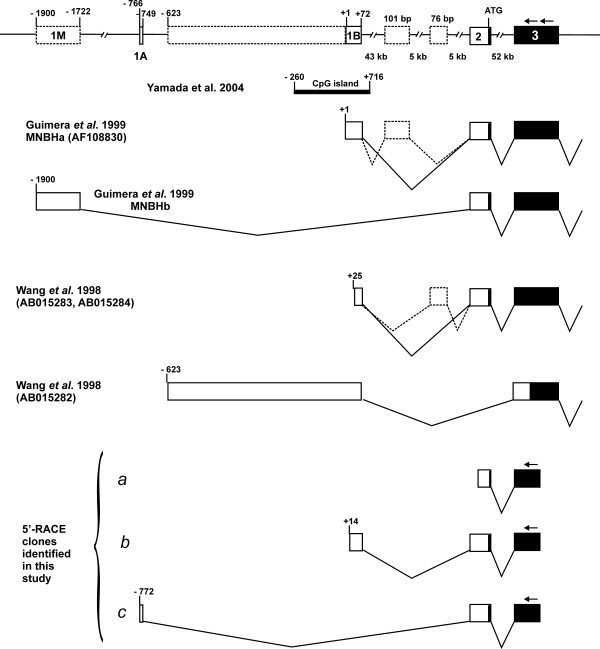
**Mapping of transcripts to the 5'-region of the human *DYRK1A *gene**. A schematic representation of the exon-intron organization is shown on top. Exons are numbered (1M, 1A, 1B, 2 and 3) and drawn to scale. Open boxes are noncoding regions, filled boxes represent the coding sequence. Open boxes with dotted lines are facultative exonic sequences that have been described in previous reports. Numbering of nucleotides in the promoter region refers to the 5'-end of transcript MNBHa which has previously been defined as the transcription start of the human *DYRK1A *gene [19] and corresponds to the transcript variant 3 (NM_101395) in the NCBI database. Position +1 corresponds to hchr21:37,661,729 in the assembly at the UCSC genome browser (release hg18). A CpG island identified by Yamada *et al*. [33] is indicated, with the bar representing the region that was analyzed in this report and found to be unmethylated. *DYRK1A *transcript variants that were previously described by Guimera et al. 1999 [19] or Wang et al. 1998 [32](database accession numbers are given in brackets) or were identified by 5'-RACE in the present study (*a, b, c*) are depicted thereunder. Positions of the primers used for RACE are indicated by arrows.

We performed RACE-PCR on cDNA from the human PC3 prostate carcinoma cell line. Sequencing of cloned PCR products revealed three types of cDNAs. The shortest clones contained cDNA fragments with their 5'-ends on exon 2 upstream of the initiator codon (transcript *a *in Figure 1). We considered these cDNAs to be truncated because several longer transcripts were already known. Second, larger products (*b *in Figure 1, represented by four RACE clones) contained cDNA fragments that started 11 nucleotides upstream of the "type 1" transcript described by Wang *et al*. [32] [GenBank:AB015283] and 14 nucleotides downstream of the transcription start of the MNBHa transcript that was previously identified by Guimera *et al*. [19] [GenBank:AF108830]. None of the RACE products from the PC3 cells corresponded to the alternatively spliced MNBHb transcript that was also described in the latter paper. However, we identified two independent clones that contained a short piece of sequence from bp -772 to -756 that was directly spliced to exon 2 (*c *in Figure 1; for detailed sequence information see Figure S1 in the Additional file 1 "supplementary figures").

**Figure 2:**
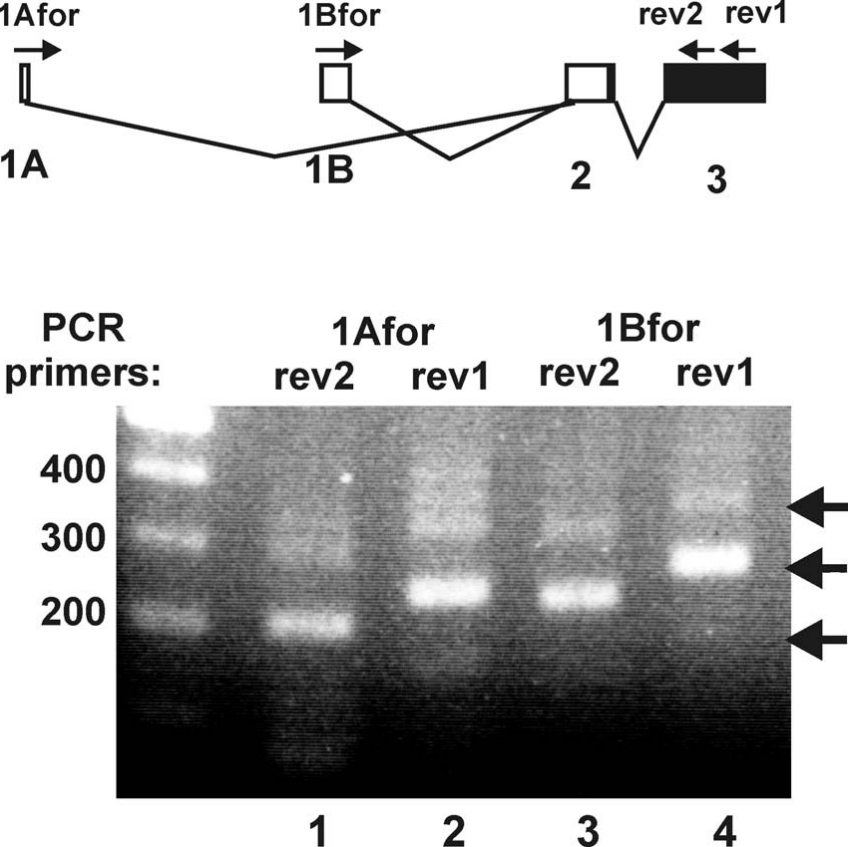
**RT-PCR analysis of the alternatively spliced *DYRK1A *transcripts**. Forward primers specific for either exon 1A (1Afor) or exon 1B (1Bfor) were used for PCR together with either one of two reverse primers targeting exon 3 (upper panel). First-strand cDNA was subjected to RT-PCR analysis with the indicated primers, and reaction products were visualized by ethidium bromide staining (lower panel). The lengths of the marker bands are indicated in bp. Expected fragment lengths are 192 bp, 232 bp, 216 bp and 256 bp for lanes 1–4. Arrows point to PCR products that were cloned and sequenced.

To confirm the existence of this alternatively spliced transcript, we performed PCR with a specific forward primers matching exon 1A and reverse primers for exon 3 (Figure 2). In parallel, a forward primer specific for exon 1B was used with the same reverse primers. We used cDNA from the human osteosarcoma cell line Saos2 as a template to exclude the possibility that the alternative transcript was an aberrant splice product specific to PC3 cells. All primer pairs yielded major amplification products that represented the expected transcripts (as indicated in the top panel of Figure 2). For each of the alternatively spliced first exons, the major bands in one of the PCRs (from lanes 2 and 4) were cloned and verified through sequencing. In addition, minor fragments were detected that were about 100 bp larger or smaller (very faint bands on the gel shown in Figure 2), and are derived from alternatively spliced transcripts (see below). The same pattern of bands was obtained by PCR on cDNA from PC3 cells (not shown).

None of the RACE or PCR products that we obtained contained the alternatively spliced additional exons between exon 1B and exon 2 that were identified by Guimera *et al*. [19] [GenBank:AF108830] and Wang *et al*. [32] [GenBank:AB015284] (see Figure 1). However, one of our RACE clones contained two additional exons between exon 2 and exon 3 (for detailed sequence information, see Figure S1 in the Additional file 1 "supplementary figures"). In addition, a clone derived from the upper band in lane 4 of the gel presented in Figure 2 contained another alternative exon between exon 2 and exon 3 (see Figure S2 in the Additional file 1 "supplementary figures"). These facultative exons are flanked by *bona fide *consensus splicing sites, but neither exon shows sequence conservation between rodents and human and they are not supported by expressed sequence tags in the database; furthermore, both contain premature termination codons. The smallest band produced by the PCR in Figure 2 represented a transcript in which exon 1B was directly spliced to exon 3, as in the clone [GenBank:AB015282] [32] (Figure 1). This transcript lacks the initiator codon on exon 2 and therefore would encode an N-terminally truncated version of DYRK1A, if another ATG codon could function as an alternative initiation codon. The relevance of these minor splicing variants is unclear.

### Structure of the DYRK1A promoter

The present results provide evidence of two transcription start sites separated by 772 bp in the 5'-region of the human *DYRK1A *gene. These start sites initiate two alternatively spliced transcripts that differ in their 5'-untranslated sequences, but are identical within their coding regions. The sequence of this region and the exact positions of the transcription start sites are shown in Figure S1 (in the Additional file 1 "supplementary figures"). We designate the alternative first exons of these transcripts exon 1A and 1B, respectively, and the corresponding transcription start sites TSS A and TSS B. Both start sites are devoid of canonical TATA or CAAT boxes, but the whole region is very GC-rich and contains numerous potential binding sites for the transcription factor Sp1 (Figure S1). TSS B lies within a CpG island that was previously found to be unmethylated in a comprehensive analysis of the methylation status of CpG islands on human chromosome 21 (#61 in Ref. [33]).

No PCR product was identified that started at TSS A and contained the whole sequence up to exon 1B. However, we cannot exclude that such transcripts escaped detection because the shorter variant might have been positively selected during PCR. TSS B is identical to the 5'-end of the MNBHa transcript described by Guimera *et al*. [19]. We designate the first exon of the MNBHb transcript proposed by Guimera exon 1M because this transcript was reported to be specifically expressed in muscle.

### Chromatin organization in the 5'-region of the human *DYRK1A *gene

Barski *et al*. [34] have recently generated a genome-wide, high resolution map of histone methylation and chromatin organization in resting human CD4+ T-cells through chromatin immunoprecipitation sequencing analysis (ChIP-Seq). The results for the *DYRK1A *gene within this dataset show that the highest level of RNA polymerase II binding was found in the region around exon 1A/1B (see Figure S3 in the Additional file 1 "supplementary figures"). Other characteristics typical of active promoters, *e.g*,. the presence of the histone H2A.Z and of histone H3 that is trimethylated on Lys4, were also specifically detected in this region. The region upstream of exon 2, which was regarded as the promoter of the *DYRK1A *gene in previous studies [35-37], exhibits neither the characteristics of an active nor an inactive promoter (*e.g*., binding of H3 trimethylated on Lys27; [34]).

### Transcription start sites of the human *DYRK1A *gene in the CAGE database

**Figure 3:**
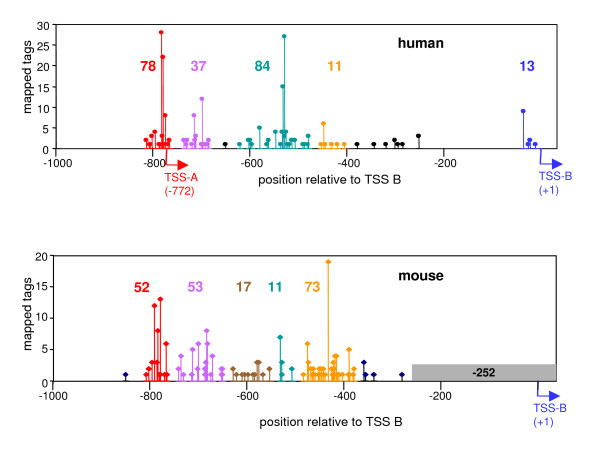
**Mapping of CAGE tags to the human DYRK1A promoter**. The CAGE database was searched for tags ascribed to the human *DYRK1A *gene (*upper panel*) and the murine *Dyrk1a *gene (*lower panel*) with the help of the CAGE basic viewer. All tags located within a region of 1,000 bp upstream of TSS B were selected, and the numbers of tags with 5'-ends at one specific nucleotide position were plotted *vs*. the position relative to TSS B. Major tag clusters (> 10 tags) as defined by Carninci *et al*. [39] are highlighted by coloring, and the number of tags forming this cluster is indicated. The region marked by a grey bar in the murine promoter was not sequenced in the genome sequence assembly (mouse May 2004) on which the CAGE tags were mapped.

To further define the heterogeneity of transcription start sites in the *DYRK1A *promoter, we took advantage of the cap-analysis of gene expression (CAGE) database of transcription start sites that was established by the FANTOM consortium [38]. CAGE tags are derived from capped mRNA molecules and thus represent the 5'-ends of individual transcripts. In total, 420 CAGE tags mapped to the human *DYRK1A *gene (transcriptional unit 723 in the database, 148,314 bp). Of these, 234 tags mapped to the 1,000 bp-region upstream of TSS B, whereas only two tags were located in the vicinity (-1,000/+100 bp) of exon 1M, and no tags mapped to the 5,000 bp upstream of exon 2. Carninci *et al*. [39] have grouped CAGE tags into tag clusters (TC) that were operationally defined to characterize promoters. As illustrated in Figure 3, major tag clusters are located close to TSS A and TSS B, respectively, confirming that the RACE clones obtained in our study represent major transcription start sites. In addition, a considerable number of tags, including the largest tag cluster of the *DYRK1A *gene (84 CAGE tags), map to the region between TSS A and TSS B. Given that each CAGE tag represents the true 5'-end of an individual mRNA molecule, this analysis shows that transcription of the *DYRK1A *gene can initiate within a broad region of about 800 bp. There was no difference in tissue distribution between the CAGE tags belonging to the different tag clusters. This unusual broad distribution of transcription start sites was also found in mouse tissues (Figure 3B), indicating that it is conserved feature of this gene.

### Promoter activities of human *DYRK1A *5'-regions

We cloned a 827 bp-sequence upstream of human exon 1M (bp -2726 to -1899), a 681 bp-region upstream of exon 1A, and a 1453 bp-region upstream of exon 1B in order to characterize their promoter activities in reporter gene assays. In addition, we generated a series of deletion constructs of the 1453-bp sequence in order to localize regulatory sequence elements and the minimal promoter region. The results of the luciferase assays in the human osteosarcoma cell line Saos2 clearly show that the two non-overlapping regions upstream of TSS A (-1453 to -756) and TSS B (-742 to +44) were able to independently drive reporter gene expression (Figure 3). Promoter region A was 14-fold more active than promoter region B, and the promoter activity of all constructs including both TSS A and TSS B was consistently higher than of those containing only TSS B. The lower activity of the -1453/+44 construct as compared to the -1453/-756 construct is possibly due to the fact that in the full length construct, transcription from TSS A proceeds through the whole region between TSS A and TSS B. Also, the presence of the unusually long 5'-UTR (which is normally spliced out from the endogenous transcripts) may impede efficient translation.

In contrast to the regions upstream of TSS A and TSS B, the putative promoter region upstream of exon 1M did not stimulate luciferase activity in Saos2 cells. The same result was obtained in COS7 cells (data not shown), and this region was not studied further in the present work. Because of the muscle-specific expression of exon 1M containing transcripts, functional analysis of the promoter region might require the use of a myoblast cell line.

**Figure 4:**
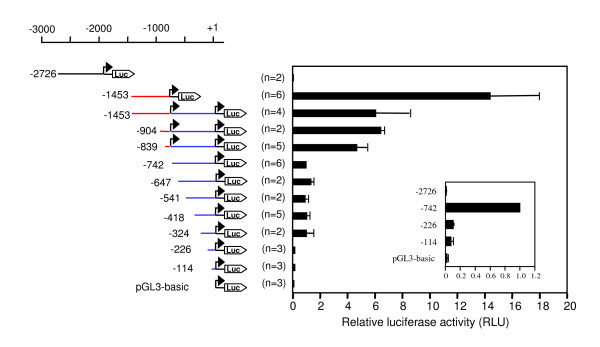
**Deletion analysis of the *DYRK1A *promoter region**. Fragments of the 5'-flanking regions of the human DYRK1A gene were fused to the firefly luciferase cDNA in the vector pGL3basic. The position of the promoter fragments relative to TSS B (+1) is indicated. Segments upstream of TSS A and TSS B are highlighted in red and blue, respectively. Saos2 cells were co-transfected with the indicated reporter gene constructs and a β-galactosidase reporter control plasmid. Cells were lysed 48 h after transfection, and luciferase activities were determined from duplicate wells. Data were normalized to β-galactosidase activities and are presented as the ratio relative to the activity of the -742 construct. The diagram integrates results of 2–6 independent experiments. Bars reflect means +/- SD. The inset shows a magnified representation of the weakest signals.

Figure 4 demonstrates that two parts of the sequence appear to be particularly important for promoter activity: Deletion of the segments between -839 and -742 or between -324 and -226 caused marked decreases of luciferase activity. However, a short segment of 114 bp upstream of TSS B was still sufficient to induce luciferase expression more strongly (3.2-fold) than the promoterless vector, and thus may be considered the minimal promoter region of TSS B.

### Upregulation of DYRK1A by E2F1

**Figure 5:**
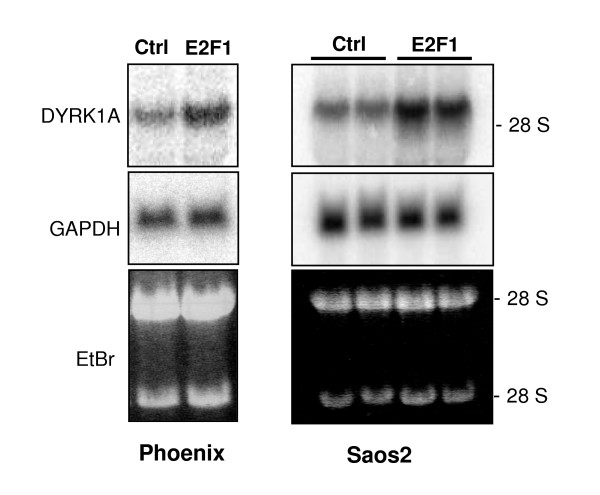
**Upregulation of DYRK1A mRNA by E2F1 overexpression**. A) Phoenix cells were transfected with an expression plasmid for E2F1 (2 μg/10 cm plate) or vector DNA (Ctrl). Total RNA was isolated 2 days after transfection and subjected to Northern blot analysis with probes for DYRK1A and GAPDH as indicated. Ethidium bromide-stained bands of the ribosomal RNAs are shown as a loading control. B) Saos2 cells expressing E2F1 under the control of a doxycycline-inducible promoter were treated for 18 h with doxycycline (E2F1), or were not treated (Ctrl) before the RNA was isolated. Duplicate lanes contain RNA from parallel plates.

In two independent microarray studies, *DYRK1A *was among the genes upregulated by overexpression of the transcription factor E2F1 in the human U2OS osteosarcoma cell line [40] and in murine NIH3T3 fibroblasts [41]. To confirm these results, we analyzed the effect of E2F1 overexpression on *DYRK1A *mRNA levels in Phoenix cells. This cell line is a derivative of human embryonic kidney cells (HEK), and was chosen for this experiment because it allows efficient transient transfection and has previously been used for analysis of E2F1 effects [42]. As shown in Figure 5A, overexpression of E2F1 in Phoenix cells caused a marked increase of *DYRK1A *mRNA levels. To confirm this result in a different cell line, we took advantage of a Saos2 cell clone that allows tetracycline-regulated overexpression of E2F1 [43]. Again, *DYRK1A *mRNA was upregulated upon induction of E2F1 overexpression.

### E2F1 specifically enhances activity of promoter region B

**Figure 6:**
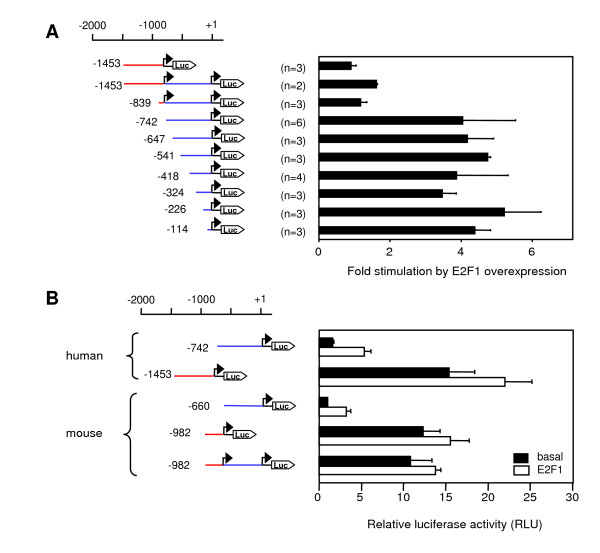
**E2F1 enhances reporter gene activity of promoter region B**. A) The inducible E2F1-expressing Saos2 cells were transfected with deletion constructs of the human DYRK1A promoter as indicated. Five hours after transfection, the medium was changed and cells were treated with doxycycline or were not treated. Data were normalized to β-galactosidase activity and are expressed as fold stimulation relative to untreated cells. The diagram integrates results of 2–6 independent experiments, and bars reflect the means +/- SD. B) Luciferase constructs of the human *DYRK1A *and the murine *Dyrk1a *promoter were analyzed for upregulation by doxycycline-induced overexpression of E2F1. Data were normalized to β-galactosidase activity and are presented as the ratio relative to the activity of the unstimulated murine -660 construct. Bars reflect the means +/- SD of 3 independent experiments.

Next we determined the effect of E2F1 on the promoter activities of the *DYRK1A *reporter gene constructs in Saos2 cells. As shown in Figure 6A, induction of E2F1 expression by doxycycline caused a four-fold increase in the promoter activity of all constructs containing only promoter region B but did not significantly affect constructs containing TSS A. Note that because of their higher basal activities (see Figure 3), constructs containing TSS A exhibited even higher promoter activities than the E2F1-stimulated constructs containing only TSS B. Therefore, the E2F1 effect was not detectable with constructs containing both TSS A and TSS B.

Next we asked whether the murine *Dyrk1a *promoter was also regulated by E2F1. For a direct comparison of human and murine promoters, reporter gene vectors including either TSS A or B were analyzed side by side in Saos2 cells. This experiment showed that the basal activities of homologous promoter constructs for TSS A and TSS B were very similar (Figure 6B). Like in the human promoter, two non-overlapping fragments of the murine promoter are capable of driving reporter gene expression, and also similar to the human promoter, promoter region A shows about 10-fold (12.3 ± 2.0 mean ± SD) higher activity than promoter region B. Overexpression of E2F1 enhanced the activity of promoter region B (-660 fragment), as was also observed with the human promoter constructs. Although these results provide clear evidence for an E2F1 effect on promoter region B, our data do not exclude the possibility that E2F1 can also affect transcription from TSS A because weak stimulatory effects may have been masked by the higher basal activity of these promoter constructs.

### Mutational analysis of putative E2F1 and Sp1 binding sites in promoter region B

**Figure 7:**
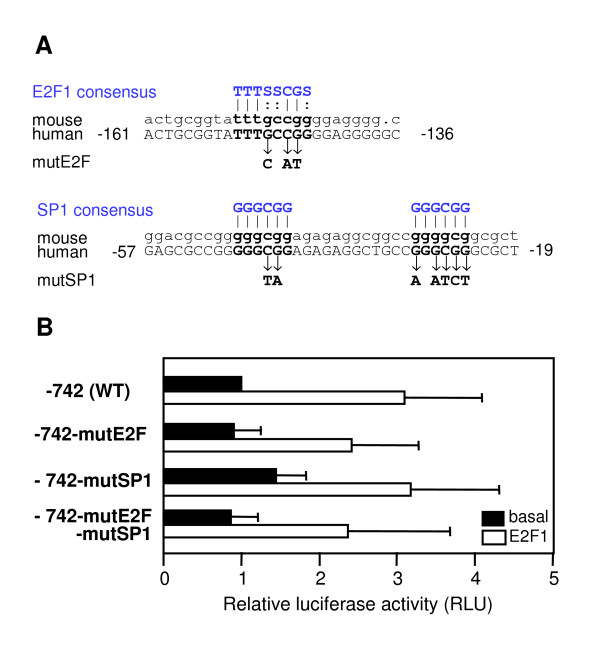
**Mutational analysis of potential E2F1 binding sites**. A) Point mutations introduced into the -742 promoter construct in order to eliminate possible E2F1 binding sites. B) The inducible E2F1-expressing Saos2 cells were transfected with the -742 promoter construct or the mutated versions thereof as indicated. Five hours after transfection, the medium was changed and cells were treated with doxycycline or were not treated. Data were normalized to β-galactosidase activity and are presented as the ratio relative to the activity of the unstimulated wild type construct. Luciferase activity of induced cells was significantly different from that in untreated cells (two-sided t test, p < 0.05, except for the double mutant) but there was no significant difference between wild type and mutants. Bars reflect the means +/- SD of 3 independent experiments.

Inspection of the 1453 bp-sequence upstream of TSS B revealed a putative E2F1-binding site at -153 that matched the criteria of a consensus sequence (Figure 7A). We generated a point mutation of this motif in the -742 reporter gene construct that is driven by the E2F1-responsive promoter B. In addition, we mutated two Sp1 sites immediately upstream of TSS B because Sp1 and E2F1 can interact in the regulation of GC-rich promoters [44-46]. Luciferase assays were performed in Saos2 cells as before to analyze potential effects of these mutations. As shown in Figure 7B, neither the basal nor the E2F1-induced promoter activity was significantly changed by the mutations. Therefore, the three sites are not essential for the regulation of TSS B by E2F1.

### Putative CREB binding site in the DYRK1A promoter

**Figure 8:**
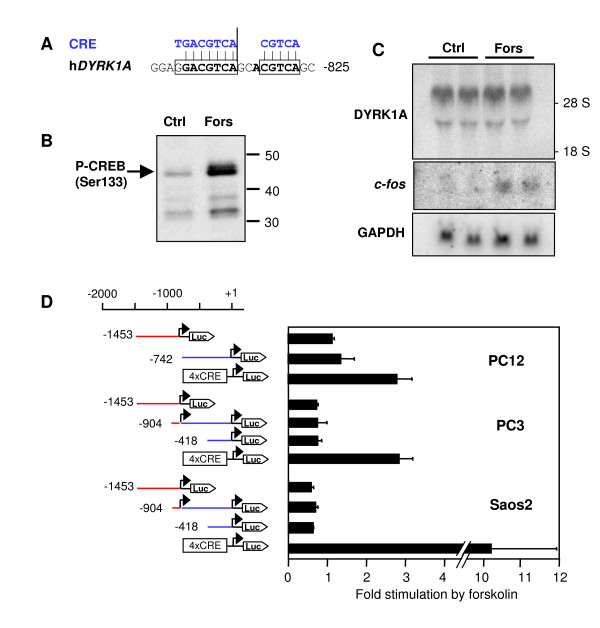
**Forskolin does not alter expression of DYRK1A**. A) Potential cAMP responsive element (CRE) upstream of TSS A. The sequence shown is fully conserved in the mouse, dog, and platypus genomes and comprises a full CRE sequence (with one mismatch to the canonical octamer) and a CRE half-site (boxed). B) Forskolin stimulates phosphorylation of CREB on Ser133. PC12 cells were treated for 30 min with forskolin (10 μM) or DMSO (Ctrl) before nuclear extracts were prepared. Phospho-Ser133 in CREB was detected by Western blot analysis with the help of a phosphospecific antibody. Migration of molecular mass standards is indicated in kDa. C) Northern blot. PC12 cells were treated for 24 h with forskolin (10 μM) or DMSO as the vehicle (Ctrl) before total RNA was isolated and subjected to Northern blot analysis with probes for DYRK1A, glyceraldehyde 3-phosphate dehydrogenase (GAPDH), and *c-fos*. Duplicate lanes contain RNA samples from parallel plates. Migration of the ribosomal RNAs is indicated in the right margin (28S, 18S). D) Luciferase assays. The indicated cell lines (PC12, PC3, Saos2) were transfected with the reporter gene constructs as schematically depicted. Luciferase activity was determined from cells treated with forskolin (10 μM) for 24 h and from untreated cells. Data were normalized to β-galactosidase activity and are presented as fold induction relative to untreated cells. Bars reflect the means +/- SD of 3 independent experiments (Saos2, PC3) or the means of duplicate wells for the experiment with PC12 cells.

In a genome-wide screen for target genes of cAMP-response element binding protein (CREB), Impey *et al*. [47], through a chromatin immunoprecipitation based method, identified *Dyrk1a *as a gene close to which CREB was bound in forskolin-stimulated rat PC12 cells. Furthermore, microarray analysis found *Dyrk1a *mRNA to be upregulated by forskolin. Because CREB is critically involved in synaptic plasticity and learning [48], we decided to study the proposed effect of CREB on the *Dyrk1a *promoter. The canonical cAMP-response element (CRE) is an eight-base palindrome (TGACGTCA) but CREs also frequently occur as half-site motifs (CGTCA) [49]. A sequence motif comprising one CRE sequence with a single mismatch and a neighboring half-site is present about 50 bp upstream of TSS A (Figure 8A). This sequence is evolutionary conserved in mammals, including mice (the sequence in rats is not yet known).

Therefore we analyzed whether *Dyrk1a *mRNA is regulated by intracellular cAMP levels. Forskolin is an activator of adenylate cyclase, and the increased cAMP levels cause the phosphorylation of Ser133, and consequently the activation of CREB by cAMP-dependent protein kinase (Figure 8B). However, treatment of PC12 cells with forskolin did not change *Dyrk1a *mRNA levels in PC12 cells, as revealed by Northern blot analysis (Figure 8C). The same result was obtained in an experiment with a PC12 clone from a different source (data not shown). As a control, a known target gene of CREB, the protooncogene *c-fos *[50], was readily induced by forskolin. In spite of this negative result, we performed reporter gene assays in order to assess the effect of forskolin on the activity of promoter region A. No stimulation of either promoter region A or B by forskolin was observed in three different cell lines (Figure 8D). In contrast, a synthetic reporter gene construct driven by four consecutive CREs was stimulated by forskolin, indicating that the cell lines analyzed were capable of a transcriptional response to forskolin/cAMP. Finally, we performed an electrophoretic mobility shift assay with nuclear extracts from forskolin-treated PC12 cells to determine whether CREB can bind to the CRE motif in promoter region A. As shown in Figure S4 (in the Additional file 1 "supplementary figures"), the probe derived from the DYRK1A gene was detected in CREB-containing complexes but the intensity of the bands was much weaker that of those with the consensus CRE.

## Discussion

*DYRK1A *has attracted considerable interest as a "candidate gene" potentially responsible for several traits related to Down syndrome. Although the characterization of transgenic mouse models has shown that the correct function of this protein kinase is critically dependent on the gene dosage and, consequently, the intracellular level of the DYRK1A protein, nothing is known about its regulation on the level of gene expression. The present study provides the first functional characterization of the human *DYRK1A *promoter.

The structural organization of the human *DYRK1A *gene has previously been described by Wang *et al*. [32] and Guimera *et al*. [19], who have also identified transcripts with 5'-ends in the promoter region. In other reports, the region upstream of exon 2 was regarded as the promoter [35-37], probably because their *in silico*-methods for promoter identification failed to correctly recognize the first non-coding exon. Of the five transcript in the NCBI CoreNucleotide database, only transcript 3 (GenBank:NM_101395) begins with exon 1B, whereas the others have 5'-ends at exon 2. The analysis of chromatin organization in the human *DYRK1A *gene (Fig. S3 in the Additional file 1 "supplementary figures") provides clear evidence that only the region around exons 1A and 1B exhibits features of an active promoter. The distribution of CAGE tags also shows no evidence of a promoter upstream of exon 2. Here we describe a new transcription start site (TSS A) which belongs to an alternative first exon (exon 1A) located 772 bp upstream of the known TSS B. Both transcript variants encode the same protein, but their expression is controlled by different promoter sequences. In addition to these two transcription start sites, evaluation of the CAGE tag database revealed that transcription can start at many more different sites within the region between TSS A and B. According to the working definition used by Carninci *et al*. [39], the promoter region of the human *DYRK1A *gene encompasses 5 non-overlapping CAGE tag clusters that by definition should represent alternative promoters (Figure 2). Each of these clusters displays a broad distribution of start sites, and according to the classification of Carninci *et al*. [39] the overall distribution can be described as "broad with dominant peaks" (-252 to -814) plus an extra cluster at TSS B (-11 to -35). It remains to be determined whether the tag clusters between TSS A and TSS B, in particular the largest cluster at about -500, represent independent core promoters.

The extended distribution of TSS is typical for promoters that lack a TATA-box and contain transcription start sites within CpG islands [39]. The region of the *DYRK1A *promoter has previously been classified as an unmethylated CpG island [33]. Consensus sites for the transcription factor Sp1 are overrepresented in promoters with high CG content, and Sp1 is capable of recruiting TATA-binding protein in the absence of TATA boxes [51]. We have studied the role of two Sp1 consensus sites close to TSS B and found that mutation of these sites did not affect the reporter gene activity of promoter region B. This result does not exclude a regulatory role of Sp1, since other Sp1 sites are present further upstream (Figure S1).

The use of alternative promoters allows for a more complex regulation of gene expression. Two different, non-overlapping fragments of the *DYRK1A *promoter region are capable of driving reporter gene expression. The higher activity of promoter region A was not specific to Saos2 cells, but was also observed in other cell lines (HeLa, COS-7, PC3, PC-12, data not shown), arguing against the possibility that the two promoters bring about tissue-specific transcriptional regulation. In accordance with this observation, the CAGE tag analysis also revealed no tissue-specificity in the composition of the different tag clusters (not shown). However, the two promoter regions differed strikingly in their response to overexpression of E2F1. All promoter constructs driven by promoter B were stimulated by E2F1, but none of the constructs that contained TSS A showed a significant response. Although promoter region A was still more active than promoter region B, total levels of *DYRK1A *mRNA were increased by E2F1 overexpression in Phoenix cells and Saos2 cells.

E2F transcription factors are well known to regulate numerous genes essential for DNA replication and cell cycle progression, and also DNA damage repair, apoptosis, development and differentiation [52]. Although so far no study has directly addressed the function of DYRK1A in the cell cycle, DYRK1A mRNA levels were repeatedly found to be regulated in systems of cellular differentiation and proliferation (see introduction). Consistent with a role in cell cycle regulation or apoptosis, DYRK1A was identified among the 150 genes whose pattern of expression in human leukemia cells most accurately discriminated responses of patients to cytostatic agents [53]. Interestingly, in transgenic mice DYRK1A overexpression has been described to be associated with high levels of cyclin B1 [54]. On the other hand, DYRK1A overexpression has been shown to enhance nerve growth factor (NGF)-mediated neuronal differentiation of PC12 cells [55]. Many E2F1 target genes including the homeobox genes are involved in developmental regulation [52], which is another area where DYRK1A has been implicated [56]. Further research is required to clarify the consequences of DYRK1A upregulation by E2F1.

Mutation of the only potential E2F binding site in front of TSS B did not affect E2F1-induced reporter gene activity. However, it is well known that some genes are regulated by E2F factors in the absence of canonical E2F sites [46,57,58]. For transcriptional activation of the human *ASK *gene, binding of E2F to a non-consensus site depends on the presence of a neighboring Sp1 site [46]. We have therefore also mutated the two Sp1 sites closest to TSS B but detected no effect on basal or E2F-induced promoter activity. Still, E2F1 can also bind to GC-rich elements in the absence of consensus E2F consensus elements, and the mutated -742 construct still contained several potential Sp1 sites [59]. Thus, our results neither exclude the possibility that E2F1 acts *via *binding to a non-consensus E2F site nor that its effect is mediated indirectly by activation or upregulation of other transcription factors.

In addition to E2F1, we tested CREB for its potential role in the regulation of the *DYRK1A *promoter because *Dyrk1a *was detected as a target gene of CREB in a chromatin immunoprecipitation-based screen in rat PC12 cells [47]. A potential CRE is located 50 bp upstream of human TSS A and is conserved in the mouse promoter. Although the motif in the *DYRK1A *promoter does not exactly match the CRE consensus sequence, it contains two copies of the CRE half site (CGTCA), which binds CREB with a lower affinity and is found in about half of the genes with functional CREs [49]. In fact, binding of CREB to this sequence in electrophoretic mobility shift assays was much weaker than binding to a consensus CRE (Figure S4 in the Additional file 1 "supplementary figures"). Forskolin did not enhance promoter activity in several cell lines, and *Dyrk1a *mRNA levels were not increased in forskolin-treated PC12 cells. Thus, it appears unlikely that CREB is a major transcriptional regulator of human or rat *DYRK1A/Dyrk1a*. However, we cannot totally exclude that the lack of a CREB effect in our hands is due to heterogeneity of PC12 subclones in different labs, or slightly divergent experimental conditions.

Up to now, the only transcription factor reported to regulate the *DYRK1A *promoter was AP4 (activator protein 4), which was described to negatively regulate expression of *DYRK1A *in non-neural cells [60]. It remains to be determined by which regulatory mechanisms, in addition to transcriptional control by E2F1 and AP4, expression levels of *DYRK1A *are controlled.

## Conclusion

The present results show that transcription of the human *DYRK1A *gene is controlled by two promoters that differ in their strength and regulation by E2F1. This finding reveals a previously unknown level of complexity in the regulation of expression of the *DYRK1A *gene, and provides the basis for further studies of its transcriptional regulation. The identification of E2F1 as a regulator of *DYRK1A *expression is consistent with the presumed function of the kinase in the regulation of cell proliferation and differentiation. Subtle changes in cell cycle regulation may affect brain development in Down syndrome, because neurogenesis involves proliferation and differentiation and is closely linked to the cell cycle [61].

## Methods

### Rapid amplification of cDNA ends (RACE)

The Marathon-Ready RACE RLM kit (Clontech, Mountain View, CA) was used for 5'-RACE of the human DYRK1A transcript on cDNA from the human PC3 prostate carcinoma cell line (Marathon-ready cDNA, Clontech). Two nested reverse primers were designed to match the second coding exon (exon 3 in Figure 1, primer sequences are given in the Additional file 2 "vector construction and oligonucleotide sequences"). PCR products were cloned, and the clones with the longest inserts were preferentially selected for sequencing.

### Database mining

The data on the chromatin structure were taken from the ChIP-Seq data generated by Barski *et al*. [34,62]. The CAGE database was searched with the help of the CAGE basic viewer [63]. Exact start positions for all tags mapped to a region of 1,000 bp upstream of TSS B were extracted from the information linked to the individual tag clusters. Analyses of the gene structure are based on the public version of the human genome [64]. Transcription start sites predicted by the eponine program [65] are also provided by the UCSC server.

### Reporter gene vectors

All human promoter constructs were obtained by PCR cloning from genomic DNA isolated from human HEK293 cells. A list of all primers and details of the cloning procedure are given in the Additional file 2 "vector construction and oligonucleotide sequences". Point mutations were introduced with the help of QuikChange™ Site-Directed Mutagenesis Kit (Stratagene, La Jolla, CA) and verified by sequencing. Murine promoter fragments were subcloned from a BAC clone (clone ID RP24-223C1 in the NCBI clone registry) into pGL3-basic using suitable restriction sites within the promoter region (*Sac*I – *Bam*HI fragment from -982 to -660, *Bam*HI-XhoI fragment from -660 to +24, numbered relative to human TSS B). As a positive control for CREB activation, we used pCRE-Luc (Stratagene, La Jolla, CA) which contains 4 copies of the CRE enhancer element (AGCCTGACGTCAGAG)_4_.

### Cell culture and transfection

The human PC3 prostate carcinoma cell line was purchased from the DSMZ (Braunschweig, Germany) (DSMZ No. ACC 465) and grown in 45% RPMI 1640/45% Ham's F12 medium supplemented with 10% fetal calf serum. Saos2 cells and Phoenix™ cells were grown in Dulbecco's modified Eagle's medium (DMEM) with high glucose (4.5 g/l) supplemented with sodium pyruvate (1 mM), L-glutamine (4 mM) and 10% fetal calf serum. PC12 cells (kindly provided by Günther Schmalzing, Institute of Pharmacology, RWTH Aachen University) were also grown in DMEM with high glucose, sodium pyruvate and glutamine but with 10% horse serum and 5% fetal calf serum. The cells from this subclone attach well on plastic dishes and form small dendrites already in the absence of nerve growth factor (NGF). A second PC12 subclone, which needed collagen coating for adherent growth and showed minimal neuronal phenotype without NGF, was obtained from Jan Tavernier (Flanders Interuniversity Institute for Biotechnology, Ghent, Belgium). Media and serum were purchased from PAA Laboratories (Linz, Austria). Transient transfections were carried out with FuGENE 6 transfection reagent (Roche Diagnostics, Mannheim, Germany) according to the manufacturer's instructions.

### Luciferase assays

Saos2 or PC3 cells were seeded in 6-well plates (200.000 cells/well) and transfected with the various luciferase vectors (0.1 μg) and 0.25 μg of the pSVβ-gal control plasmid (Promega, Madison, WI, USA). Two days after transfection, cells were washed in PBS and then harvested in reporter lysis buffer (Promega). PC12 cells were seeded at a density of 600.000 cells/well. In some experiments, forskolin (10 μM; Merck-Calbiochem, Darmstadt, Germany) or vehicle (dimethyl sulfoxide) was added one day after transfection for 24 h before cell lysis (Figure 8). For experiments involving induction of E2F1, doxycyclin (2 μg/ml) was added 5 h after transfection, and cells were lysed 17 h later.

Luciferase activities were measured from duplicate wells with the help of a commercial kit (Promega) and data were normalized to β-galactosidase activities. β-galactosidase activities were determined by the luminescent galactosidase detection kit II (Clontech). The protein concentration of the cell lysates was measured using the BCA protein kit (Pierce, Rockford, IL, USA).

### Overexpression of E2F1

A plasmid for transient mammalian expression of untagged, full length human E2F1 (pCMV-SPORT6) was purchased from the Resource center/Primary Database (RZPD, Berlin) ([GenBank:BC050369], clone IMAGE:6025053). A stably transfected Saos2 cell clone expressing E2F1 under the control of a tetracycline-regulated promoter [43] was kindly provided by Karen Vousden (Beatson Cancer Institute, Glasgow, UK).

### Northern and Western blot analysis

Total RNA from cells cultured in 10 cm-plates was isolated using the RNeasy Midi Kit (Qiagen, Hilden, Germany). Samples (15 μg) of total RNA were separated by denaturating formaldehyde electrophoresis on 1% (w/v) agarose gels and transferred by capillary blot onto positively charged nylon membranes (Hybond N+; Amersham, Freiburg, Germany). As the probe for DYRK1A, a 1,400 bp *EcoR*I fragment from the 5'-end of the human DYRK1A cDNA was labeled with ^32^P by random oligonucleotide priming. After probe removal by incubation with 0.5% SDS at 100°C, blots were rehybridized with a probes specific for glyceraldehyde 3-phosphate dehydrogenase (GAPDH) (kind gift of Claudia Krusche, Aachen) and *c-fos*. To generate the template for a *c-fos*-specific probe, the cDNA encoding the open reading frame of rat *c-fos *was amplified with specific primers, cloned and sequenced.

For Western blot analysis of CREB phosphorylation, nuclear extracts were prepared from PC12 cells by hypotonic lysis [66]. Forskolin was added at a final concentration of 10 μM to some of the samples as indicated in the figure legend. For Western blot analysis, protein samples were separated by SDS-PAGE (10% gel), blotted onto nitrocellulose, and phosphorylation of CREB on Ser133 was detected by chemiluminescence using a phosphospecific antibody (Upstate, Charlottesville, VA) and horseradish peroxidase-labeled secondary antibody (Pierce, Rockford, IL).

## Authors' contributions

BM carried out most of the experiments. PH devised conditions for the EMSA experiments. EMV cloned the murine *Dyrk1a *promoter and constructed the murine reporter gene vectors. JG participated in the design of the study and final editing of the manuscript. WB conceived of and planned this study and wrote the manuscript. All authors read and approved the final manuscript.

## Supplementary Material

Additional file 1 **Supplementary figures**. This PDF file contains four additional figures which show the sequence of the promoter region of the human *DYRK1A *gene (Fig S1), the sequences of two transcripts with additional exons between exon 2 and 3 (Figure S2), the ChIP-seq data from the study of Barski *et al*. [34] for the 5'-region of the human *DYRK1A *gene (Figure S3), and the result of an electrophoretic mobility shift assay (EMSA) showing weak binding of CREB to the CRE motif in promoter region A (Figure S4). Click here for file

Additional file 2 **Vector construction and oligonucleotide sequences**. This PDF file includes a detailed description of the construction of the reporter gene vectors and a complete list with the sequences of the oligonucleotides used in this study. Click here for file
