# Supplementary material for: Characterization of the human DYRK1A promoter and its regulation by the transcription factor E2F1
Source: BMC Mol Biol. 2008 Mar 26;9:30. doi: 10.1186/1471-2199-9-30 (PMC2292204; doi:10.1186/1471-2199-9-30)
Supplement: Additional file 1 — Supplementary figures. This PDF file contains four additional figures which show the sequence of the promoter region of the human DYRK1A gene (Fig S1), the sequences of two transcripts with additional exons between exon 2 and 3 (Figure S2), the ChIP-seq data from the study of Barski et al. [34] for the 5'-region of the human DYRK1A gene (Figure S3), and the result of an electrophoretic mobility shift assay (EMSA) showing weak binding of CREB to the CRE motif in promoter region A (Figure S4). [file 1471-2199-9-30-S1.pdf]

Additional file 1: Supplementary Figures

This PDF file contains four additional figures which show the sequence of the promoter region of the human *DYRK1A* gene (Fig. S1), the sequences of two transcripts with additional exons between exon 2 and 3 (Fig. S2), the ChIP-seq data from the study of Barski *et al*. [34] for the 5‘-region of the human *DYRK1A* gene (Fig. S3), and the result of an electrophoretic mobility shift assay (EMSA) showing weak binding of CREB to the CRE motif in promoter A (Fig. S4).

```
                      -1453
1470 aggaggcccgccagcccttcgaagaaccggctccacgcgcggacggtcgcggccgccgcttcccgggg
1400 agagaggcgagcgcggcgcccggaccgcggtgagagccctccaggcccgggcgcgcggggtgggcttccc
1330 gggtccccgcccgaaggccgcggcgcccctcctcctcccctcccccgcccggggcagcccgccccctccc
1260 cccggcgcgggcggggctgcgggcgccgacgcggaagcgcggggcgctagggctgcggggcggcggcgc
1190 gggaggccgcgcgcgctccccgggccgcagtcggcgcgggcgcggggcggggcggggcggccgggaggcg
1120 gcgggcgatcgcgcacaggcctggccgccgcctcgcccccccttccccagcccctaccctccctcggcct
1050 agccgtccccctcctctcccctcccccgggcgaatcacgcgccctcctctgacactcgtagcggacccga
-980 gctccgaatttatccttcacgtgacctgaggggttgggtggttggctggggacagaggggagacggaa
         -904
-910 aggagggaacggaatctgccgttacctgagcaacaggccccgcccgccggctcggggctgggggcggga
      -839                 CRE                                           TSS A
-840 ggaggacgtcagcacgtcagccggggttttgcgttttgattgacagttgctatagcgaccgggtcggtCC
                                 -742
-770 GTCGCCATTTTGTTGgttggtttttttttaaaccctttcgcttcccgcccgaataataataaaaagcccc
                                                                 -647
-700 attggagtgaggcggggggtggcggcggcaaccgcggcggggggtatccggggagactgctgctgtcgctgc
     AP4      AB015282
-630 tgctgatcgcggcccaggtcggcctcagagagcggacaccccgagcggggggtgcgggcgccgccgccgc
                      -541
-560 cgcttctgctgctgctgttcctgctgctgctgttggtgccgctgccgccgccggcgagcaggcgggaccg
-490 gtgtgagtgcgagtgtgtgtgcgagggagcgtgtgcgagggcgattgtgcgcgcgagtgtgcgggtgtgt
      -418
-420 gcgagtgtctgtctgtctgtgcgggactcgggggcgggcggttcgggctctcctggcggaggagccgcc
                                 -324
-350 gccgccgcccgggcccggcgcttctcgctgcgcaggttcggagcgcgcgccatttttgtgagatttacaa
                                                                  -226
-280 aatcctcctcgggaagaagccgccggcagcagccgccgctcggcgcccggcctcgccgacgccgcccctct
               g                                               E2F1
-210 gcgccgggccggccgtgcggccccgccgcctggaatcgggcccggggactgcggtatttgccggggagg
                                 -114
-140 gggctgtcgcctccccggccccgggcgccgctggaaccgcgagccgaggagagactgagcaggctgcggc
 -70 gcggccaggagccggagcgccgggggcggagagaggctgccggggcggcgctggctgcggaggccgcggc
TSS B  AF108830                 AB015283
  +1 GGGAGCGCGGCGCGGGAGCCCGAGGCTGAGACTCACCGGAGGAAGCGGCGCGAGCGCCCCGCCATCGTCC
 +71 CGgtgagtgtccggcccggccgcggccccgcccggcctggcgctcggctcccccggcggcccgcagccgg
```

Figure S1: Promoter region of the human *DYRK1A* gene

The sequence was taken from the UCSC Human Genome Browser (hg18, assembly March 2006) [64]. The sequence of the promoter constructs that was amplified from HEK293 cells contains an additional G at position -201 (*triangle*) that is absent in the UCSC genome assembly (insertion/deletion polymorphism refSNP ID:rs34481457). For sake of consistency with the previous report by Guimera *et al.* [19], we define the first nucleotide of the MNBHa transcript (GenBank acc. AF108830) as the transcription start of exon 1B (numbered +1, corresponding to hch21:37,661,729 in hg18). Exon 1A and exon 1B are shown in *red* and *blue*, respectively. The 5'-ends of transcripts described in previous papers are indicated (AF108830, AB015282, AB015283). Putative binding sites for the transcription factors E2F1, CREB, and AP4 are *boxed*; and Sp1 consensus sites are highlighted by *shaded boxes*. The 5'-ends of the deletion constructs are flagged by numbers referring to the position of their first nucleotide as in the figures 4, 6, and 8.

RACE clone G7

```
         37714556 (exon 2)
         ATCAGGATGATATGAGACTTGAAAGAAGACGATGCATACAGgtgac....
                                         M  H  T
                   37719863
...tcccattttcctctagAAGAGACAGAGTAACTAGCTCAGCAAAACCAAAACGTCAC
                   E  E  T  E Stop
ATTCAGCATCTGCAAGACTAGGTGAACAGTATCCCCACTGACTCACATACAAgtaag..
                                        37719957

                   37724441
...tttattttgtcttagAATCTTCTGAATATTGAAAACAGAGAACTATACTGGAAGA
ACATAGTGTATTAAGACTCATGGAGAGGGAGATGTGATACTGTGTCACTGAGGTCGTTC
CAGTCATAGGAGAAATGTTACCACTGGATTGAGgtctggtacatttta...
                         37724572

                         37766856 (exon 3)
..tcacttatcttcttgtagGAGGAGAGACTTCAGCATGCAAACCTTCATCTGTTCGGC
TTGCACCGTCATTTTCATTCCATGCTGCTGGCCTTCAGATGGCTGGAC
                           <--------------------
                            primer rev2
```

PCR product from the last lane in Figure 2 (upper band)

```
37661748 (exon 1B)                           37661802
CCGAGGCTGAGACTCACCGGAGGAAGCGGCGCGAGCGCCCCGCCATCGTCCCGgtgagt
---------------->
    primer 1Bfor
                   37714471  (exon 2)
tctgttttttcttcacacagTGTTATAGTTTTGCCGCTGGACTCTTCCCTCCCTTCCCCC
ACCCCATCAGGATGATATGAGACTTGAAAGAAGACGATGCATACAGgtgactcaagt
                                        M  H  T

                   37715946
tttgacctttttggtgcagGAACTATTTCTCAGCATTGTCAGCTCCTGGATTGCTCCTTG
                   G  T  I  S  Q  H  C  Q  L  L  D  C  S  L

GGCGCTATACTGCACATTGGCTATACCAAGTAGATGCTCACTTTCGACTGAGgtcagta
 G  A  I  L  H  I  G  Y  T  K Stop          37716038

                   37766855 (exon 3)
cctcacttatcttcttgtagGAGGAGAGACTTCAGCATGCAAACCTTCATCTGTTCGGC
TTGCACCGTCATTTTCATTCCATGCTGCTGGCCTTCAGATGGCTGGACAGATGCCCCAT
TCACATCAGTACAGTGACCGTCGCCAGCC
   <-------------------------
           RACE primer rev1
```

Figure S2: Transcripts with additional exons between exon 2 and 3

Two cDNAs of human *DYRK1A* were identified in our study which contained alternative exons between exon 2 and exon 3. The intron/exon borders fulfil the minimal requirements for splicing sites (GT..AG and the pyrimidine-rich stretches are *underlined*). However, both transcripts contain termination codons shortly after the start, and neither of these facultative exons is evolutionary conserved or is represented by ESTs.

Exonic sequences are shown in capital letters, the coding sequence is shown in bold print, and the additional exonic sequences are shown in blue. Numbering refers to the first and last nucleotides of the exonic sequences.

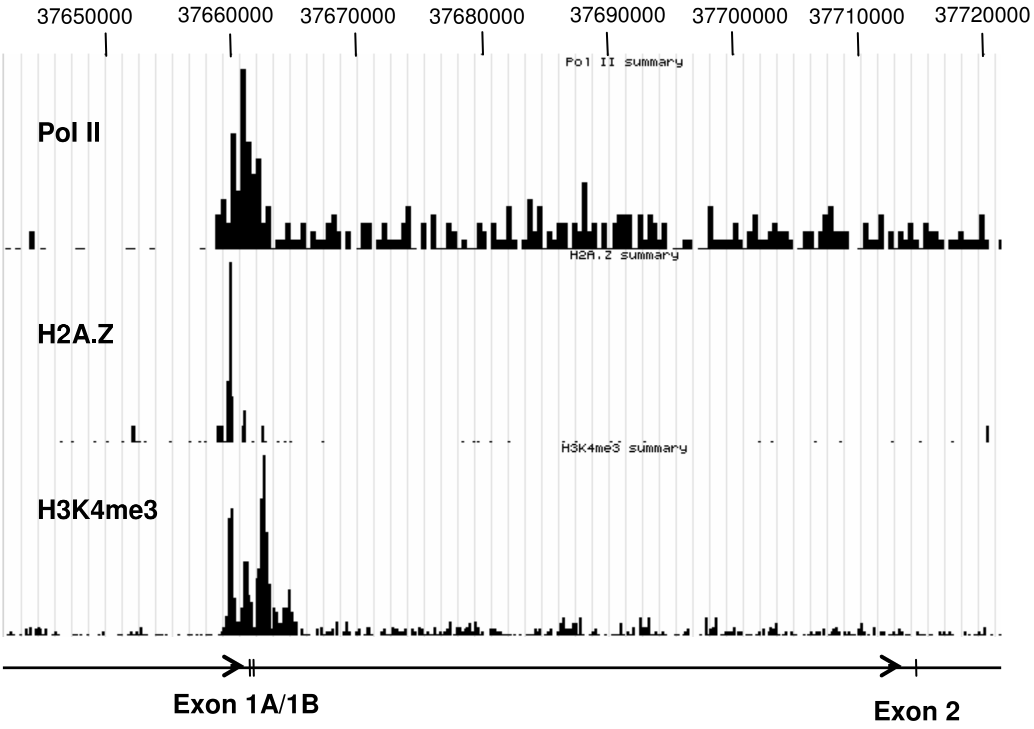

Figure S3: Chromatin structure in the 5'-region of the human *DYRK1A* gene

A high resolution map of histone methylation and chromatin organization in resting human CD4+ T-cells was recently generated by Barski *et al.* [34, 62] using chromatin immunoprecipitation sequencing analysis (ChIP-Seq). The maps present the data obtained in this study and illustrate binding of RNA polymerase II (Pol II), histone H2A.Z, and Lys4-trimethylated histone H3 (H3K4m3) to the 5'-region of the human *DYRK1A* gene. The positions of exons 1A, 1B and exon 2 are indicated in the bottom. Arrowheads indicate the promoter region in front of the exons 1A and 1B, which was studied in the present paper, and the region before exon 2, which was regarded as the *DYRK1A* promoter in previous studies (from 37,712,426 to 37,713,112 in Ref. [36]; from 37,712,205 to 37,712,787 in Ref. [37]).

The presence of the Pol II island at exon 1A/1B is characteristic of a promoter of an actively transcribed gene. H2A.Z and H3K4me3 are also enriched in promoter regions close to the TSS and are positively correlated with gene expression.

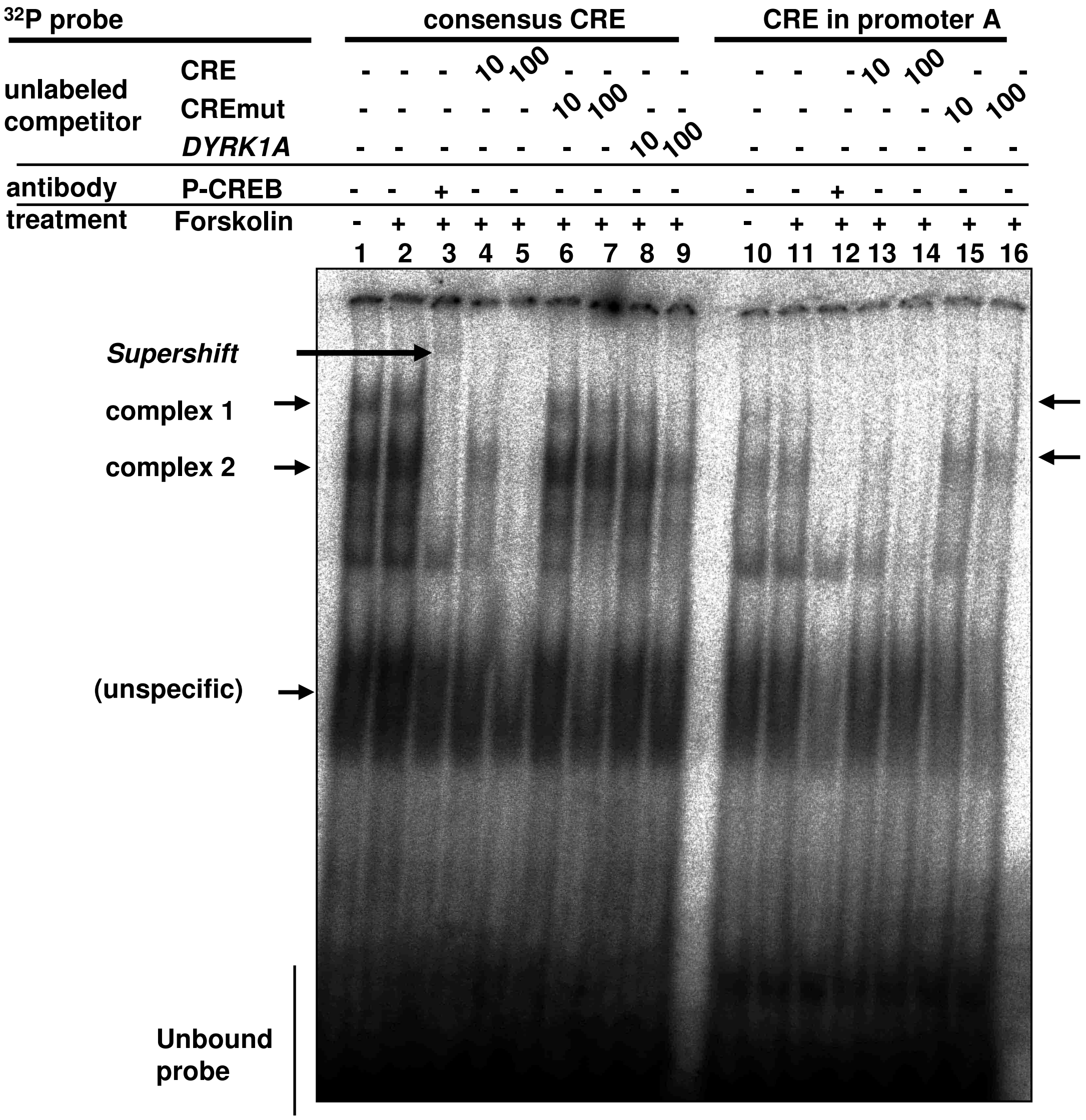

Figure S4: Weak binding of CREB to the CRE-like motif in promoter A

Nuclear extracts were prepared from PC12 cells after treatment with forskolin (10 µM for 30 min) or from untreated cells (lane 1). Samples were subjected to gel retardation assays with radioactively labeled probes harboring the octameric consensus CRE (**TGACGTCA**) or the CRE-like motif from the human *DYRK1A* promoter (G**GACGTCA**GCA**CGTCA**). Unlabeled competitor probes were used at a 10-fold or 100-fold molar excess. CREmut differed by two bases from the consensus CRE (**TG**TG**GTCA**).

Two complexes were detected with the consensus CRE and were dose-dependently competed by an excess of the unlabeled probe (lanes 4 and 5) but not by the probe with the mutated CRE (lanes 6 and 7). Both complexes disappeared when a pSer133-specific antibody was included in the binding reaction (lane 3). The intensity of both complexes was also reduced by competition with the probe from the *DYRK1A* promoter, although a 100fold excess was required to achive the same effect as with a 10fold excess of CRE. The ^{32}P-labeled probe from the *DYRK1A* promoter produced weaker bands of complexes 1 and 2. These bands were also competed by the CRE probe (lanes 13 and14), but not by CREmut (15 and 16), indicating that they contain CREB.
