# Supplementary material for: Characterization of the human DYRK1A promoter and its regulation by the transcription factor E2F1
Source: BMC Mol Biol. 2008 Mar 26;9:30. doi: 10.1186/1471-2199-9-30 (PMC2292204; doi:10.1186/1471-2199-9-30)
Supplement: Additional file 2 — Vector construction and oligonucleotide sequences. This PDF file includes a detailed description of the construction of the reporter gene vectors and a complete list with the sequences of the oligonucleotides used in this study. [file 1471-2199-9-30-S2.pdf]

Additional file 2:
Vector construction and oligonucleotide sequences

Construction of reporter gene plasmids

All human promoter constructs were obtained by PCR cloning from genomic DNA isolated from the human HEK293 cell line. Amplification of the highly GC-rich template was achieved by using the JumpStart REDAccuTaq LA polymerase mix (Sigma-Aldrich) and adding DMSO to the reaction mix at a final concentration of 9%. The reporter construct for the putative promoter in front of human exon 1M (TSS-M) contained bp –2726 to –1899. Promoter constructs for TSS A and TSS B contained fragments of variable length as indicated in the figure legends. Suitable restriction sites were introduced by the design of the PCR primers. PCR products were cloned between the *Nhe*I-site and the *Bgl*II-site (exon 1M) or between the *Nhe*I-site and the *Hin*dIII-site (all other human constructs) in front of the firefly luciferase cDNA in the vector pGL3-basic (Promega, Mannheim, Germany). Sequencing of several independent clones revealed the presence of an additional G between bp –200 and -201 in comparison with the human genome sequence accessible at the UCSC Genome Bioinformatics Site (genome.ucsc.edu; release hg18). This difference is a known insertion/deletion polymorphism (refSNP ID:rs34481457). In addition, the following differences from the database sequence were identified: construct –1453, T(-938)C, construct -904, C(-676)T, construct –418, G(-179)A and C(-13)T. Construct -1453 was used as a template to amplify the other deletion fragments.

Sequences of the oligonucleotides used for PCR and as EMSA probes

Oligonucleotides used as primers for RACE and for PCR in Fig. 2

Primer	Sequence 5´ → 3´	
Rev1<br>Rev2	GGCTGGCGACGGTCACTGTACTGATG<br>GTCCAGCCATCTGAAGGCCAGCAG	Exon 3<br>Exon 3
1Afor<br>1Bfor	AATTGCTAGCGTCCGTCGCCATTTTGTTG<br>CCGAGGCTGAGACTCAC	Exon 1A<br>Exon 1B

Oligonucleotide primers for cloning of promoter constructs

Position relative to TSS-B	Sequence 5´ → 3´	Restriction site
-2726for<br>-1899rev	AATTGCTAGCTTGGTTGTATAGGTGGAAGC<br>AATTAGATCTCAGAGTCTTAATTCGCCTGC	*Nhe*I<br>*Bgl*II
-1453for<br>-756rev	AATTGCTAGCTTCGAAGAACCGGCTCCACG<br>TTAAAAGCTTCAACAAAATGGCGAC	*Nhe*I<br>*Hind*III
-904for<br>-839for<br>-742for<br>-647for<br>-541for<br>-418for<br>-324for<br>-226for<br>-114for<br>+44rev	AATTGCTAGCGAACGGAATCTGCCGTTACC<br>AATTGCTAGCGAGGACGTCAGCACGTCAGC<br>AATTGCTAGCTAAACCCTTTCGCTTCCCGC<br>AATTGCTAGCACTGCTGCTGTCGCTGCTGC<br>AATTGCTAGCCCTGCTGCTGCTGTTGGTGC<br>AATTGCTAGCGAGTGTCTGTCTGTCTGTGC<br>AATTGCTAGCCGCTGCGCAGGTTCGGAGC<br>AATTGCTAGCGCCGACGCCGCCCTCTGC<br>AATTGCTAGCGCCGCTGGAACCGCGAGC<br>AATTAAGCTTTTCCTCCGGTGAGTCTCAGC	*Nhe*I

Oligonucleotides used for site directed mutagenesis:

	Sequence 5´ → 3´	
Mutation of<br>E2F1 site	GGACTGCGGTATTTCCATGGGAGGGGGCTGTC<br>GACAGCCCCCTCCCATGGAAATACCGCAGTCC	*Nco*I
Mutation of<br>Sp1 sites	GCGCCGGGGGTAGAGAGAGGCTGCCAGATCTGCGCTGGCTGCG<br>CGCAGCCAGCGCAGATCTGGCAGCCTCTCTCTACCCCCGGCGC	*Bgl*II

Oligonucleotides used for EMSA probes:

Name of probe	Sequence 5´ → 3´
Consensus CRE	AGAGATTGCC**TGACGTCA**GAGAGCTAG<br>CTAGCTCTC**TGACGTCA**GGCAATCTCT
Mutated CRE	CAGGTAGATC**TG**TG**GTCA**AGAGATGGC<br>GCCATCTCT**TGAC**CA**CA**GATCTACCTG
CRE-like motif<br>in promoter A	GGCGGGAGGAG**GACGTCA**GCACGTCAG<br>CTGACGTGC**TGACGTC**CTCCTCCCGCC
